# Age Matters: A Study on Perceived Discrimination Among Older Adults in Healthcare in Lithuania

**DOI:** 10.3390/healthcare13243192

**Published:** 2025-12-05

**Authors:** Kristina Selli, Ramunė Kalėdienė, Skirmantė Sauliūnė, Mindaugas Stankūnas, Snieguolė Kaselienė

**Affiliations:** Department of Health Management, Faculty of Public Health, Lithuanian University of Health Sciences, 47181 Kaunas, Lithuania; ramune.kalediene@lsmu.lt (R.K.); skirmante.sauliune@lsmu.lt (S.S.); mindaugas.stankunas@lsmu.lt (M.S.); snieguole.kaseliene@lsmu.lt (S.K.)

**Keywords:** ageism, older adults, healthcare access, discrimination in healthcare, patients’ rights, health equity, Lithuania

## Abstract

**Objective**: The aim of this study was to analyse the opinions and experiences of older adults regarding age-based discrimination in healthcare. **Method**: The study is based on the analysis of data from 492 older adult respondents aged 60–84, with the mean age of 71.6 ± 6.6 years, who completed and returned anonymous questionnaires sent to their homes. **Results**: Most of the respondents (74.8%) believed that the state does not consistently improve the protection of older persons’ rights. Nearly half (42.5%) reported that healthcare services are not provided equally to younger and older people. A significant proportion of respondents (41.1%) reported experiencing age-based discrimination themselves, having responded affirmatively to at least three out of nine statements indicating discriminatory experiences. Poor health status and financial hardships emerged as the primary factors associated with multidimensional discrimination experienced by older adults. More than half (64.0%) of respondents believed that discriminatory attitudes towards older people are rooted in healthcare professionals’ internal cultural norms. **Conclusions**: The findings of the study indicate the need to change the attitudes of healthcare professionals towards older adults. There is an urgent need for the targeted professional education on this issue.

## 1. Introduction

### 1.1. Background on Ageism

Ageism is prejudice or discrimination based on age [1]. Ageism manifests in various areas of life, including healthcare. It represents a barrier to healthy ageing [2]. Decades after its conceptualisation, the World Health Organization (WHO) warns that ageism is widespread globally and “associated with earlier death” and poorer physical and mental health among older adults [3]. This is borne out by large reviews: Chang et al. found that in 95.5% of studies ageism was linked to worse health outcomes across 45 countries [4]. Likewise, Allen et al. reported that nearly all (93%) United States (U.S.) older adults experienced everyday ageism, which significantly increased their odds of depression, functional limitations and other negative outcomes [5].

More importantly, ageism is linked to poorer health and care outcomes. According to Chang et al. (2020), most studies found that ageist attitudes led to significantly worse health across mental, physical and social domains [4]. In Allen et al.’s survey, higher everyday ageism scores raised the odds of depressive symptoms and functional decline (e.g., a one-point increase in the ageism scale raised depression odds by 20%) [5]. Ageism can also deter care-seeking: a U.S. survey found that ~23% of older adults avoided medical care, often due to fear, discomfort or distrust in providers [6]. These patterns suggest that prejudices and stereotypes at least partly explain why some older adults under-utilise needed services and experience worse health.

### 1.2. Existing Evidence and Current Challenges

Recent studies document how ageism appears in health settings. A scoping review of 41 studies identified two main expressions of health-care ageism: at the interpersonal level, through demeaning attitudes and neglect of older persons’ needs; and at the institutional level, through barriers to care and neglectful practices [7]. Liu et al. (2024) identified four dimensions of perceived ageism in healthcare–self-internalisation (patients’ own feelings), interpersonal biases (negative attitudes and unfair treatment), organisational factors (unfamiliar technology, unsupportive environment, lack of resources), and policy issues (one-size-fits-all procedures) [8]. Allen et al. (2022) distinguished three forms of “everyday ageism” (internalised, interpersonal, and exposure to negative messages), all of which were highly prevalent and correlated with worse health [5]. In sum, the literature portrays ageism as a multi-faceted problem in healthcare ranging from overt neglect to subtle negative assumptions about ageing.

Studies have examined which groups of older adults are most affected by ageism. Allen et al. (2022) found that adults aged 65–80 reported higher ageism scores than those 50–64, and those White and Hispanic respondents experienced ageism more frequently than Black respondents [5]. Allen et al. (2023) found that older age, lower education and lower socioeconomic status predicted higher reported ageism, whereas findings for sex and race/ethnicity were mixed [9]. In another study researchers found that perceived discrimination was higher among migrants from low-income countries and among women [10]. Yet another study reports a completely different conclusion, namely that in most countries, people with higher education, older people, women and the indigenous population reported feeling less discriminated against [11]. Such analyses imply that both individual characteristics and structural factors shape how age discrimination is experienced.

Furthermore, ageism in healthcare intersects with broader policy and human rights issues. The United Nations has stressed that no one should be “left behind” in achieving Sustainable Development Goals, explicitly highlighting inclusion of older persons in health and social policy [12]. The WHO has launched global campaigns to combat ageism, calling for evidence-based strategies at individual and institutional levels [13]. Yet legal protections remain uneven. In Europe, European Union (EU) law prohibits age bias in employment but until recently it provided limited coverage of discrimination in healthcare or services [14]. The European Court of Human Rights (ECHR) recognises age as a prohibited ground (“other status”), but its case law has generally allowed age-based distinctions if framed as serving a “legitimate aim” [14]. For example, ECHR rulings have upheld upper-age limits for housing benefits or adoption rights when justified by demographic or child-welfare goals [14]. Thus, legal authorities have been reluctant to treat ageism on par with sex or race discrimination [14].

The emerging research indicates that digital technologies (DT) contribute to the intensification of age-based discrimination: “study among physiotherapists in Luxembourg demonstrates for the first time that DT-based ageism can lead to discrimination and deprive older persons of receiving optimal physiotherapy treatment” [15]. Consequently, the relevance of this topic is therefore likely to persist for a long time.

There is a growing consensus that addressing ageism will require multi-level solutions. In an earlier qualitative study conducted by the authors of this article, the need for formal policies and patient rights protection was emphasized: for instance, studies of Lithuanian older adults identified barriers such as the lack of legal recourses and awareness, and recommended new laws, complaint systems, and rights-based education to empower older patients [16]. In a global context, combating ageism is now framed as essential for equity and healthy ageing [12].

### 1.3. Rationale and Significance of the Study 

This field still lacks sufficient research. Ayalon and Tesch-Römer, in their book, emphasize that quantitative studies of ageism using surveys for data collection are almost exclusively directed at care professionals, rather than care users. They also highlight that data are typically collected in a single institution, often based on a specific disease, and that existing ageism scales are not related to healthcare systems [17]. Given the substantial international evidence documenting ageism and its correlates in healthcare, it is crucial to establish whether Lithuania exhibits similar or distinct patterns. To our knowledge, no quantitative studies have examined age-based discrimination within Lithuanian healthcare. The literature review did not identify quantitative studies examining how older adults’ own personal characteristics or experiences of age discrimination relate to their perception of the causes of such discrimination. Instead, the available evidence consists predominantly of qualitative research focusing on healthcare providers’ attitudes, rather than on the perspectives of older adults themselves. In this study, older adult respondents—users of healthcare services—were surveyed to explore potential associations between their personal characteristics and experiences and their perceptions of the factors contributing to age-based discrimination. This study therefore fills a clear gap by providing the first systematic data on older adults’ experiences of ageism in Lithuania, enabling direct comparison with findings from other countries and investigation of how structural, cultural, and service-level factors may differentially shape discriminatory practices. These novel data will both situate Lithuania within the broader cross-national literature and inform locally tailored policy and practice responses.

### 1.4. Study Objectives and Hypothesis 

This study aims to analyse the perceptions and experiences of older adults in Lithuania regarding age-based discrimination in healthcare. By examining the extent of perceived ageism, the research seeks to identify key factors contributing to discriminatory practices and inform about the strategies to mitigate their impact. The findings will provide scientifically grounded evidence of perceived age-based discrimination in healthcare, the dissemination of which is expected to inform and influence health policy development in Lithuania, while also contributing to the broader international discourse on ageism in healthcare.

Specifically, the authors aim to identify how frequently such discriminatory experiences occur and which manifestations are most common from the perspective of older patients. The hypothesis is as follows: perceptions of age-based discrimination in Lithuania’s healthcare system among older adults are associated with their sociodemographic characteristics (age, gender, education, financial and health status), the internal culture of healthcare institutions, and perceptions of national policy orientation.

## 2. Methods

### 2.1. Study Design and Population

This study is a part of a cross-sectional research project conducted to characterise the implementation of older adults’ rights in healthcare. A stratified random sample of 1300 individuals aged 60–84 years was selected from the population of Kaunas city using data provided by the Centre of Registers under the authority of the Government of the Republic of Lithuania. The sampling strategy included an intentional over-representation of the oldest age group, achieved through age-stratified randomisation. Prior to data collection, a minimum required sample size of 387 was calculated using Raosoft sample size calculator (Raosoft Inc., Seattle, WA, USA) to ensure adequate statistical sample. Data were collected during the summer of 2019 using an anonymous questionnaire sent by post to participants’ home addresses. A total of 492 completed questionnaires were returned, yielding a response rate of 37.85%. The research protocol was approved by the Kaunas Regional Biomedical Research Ethics Committee (No. BE-2-81).

### 2.2. Tools and Measurements

#### 2.2.1. Age Discrimination in Healthcare

Older adults’ attitudes to age discrimination in healthcare were assessed by asking nine questions. The first two questions assessed respondents’ attitudes towards state-guaranteed healthcare and policy: “Does the state guarantee equal healthcare for all age groups (both young and old)?” and “Does state health policy consistently improve the protection of the rights of older people in healthcare?” with answers “Yes” (coded as 1), “No” (coded as 0), “I don’t know” (those, who answered “I do not know”, were eliminated from the analysis). These two questions were reverse-coded. The next seven questions/statements were designed to assess the respondents’ experiences of age discrimination in healthcare institutions: “The older a person is, the less their needs are met in healthcare and healthcare policy” (coded as “Yes” = 1 vs. “No” (“Needs met the same”, “Needs met more”) = 0); “The older a person is, the less attention and care doctors/other healthcare professionals show” (coded as “Yes” = 1 vs. “No” (“Shows the same amount of attention and care”, “Shows more attention and care”) = 0); “The older a person is, the more doctors/other healthcare professionals delay providing help” (coded as “Yes” = 1 vs. “No” (“Provides help the same”, “Does not delay providing help”) = 0); “Have you felt that you were treated worse by healthcare staff because of your age?”, “Have you been discouraged from procedures, surgeries or other services because of your age” and “Have you been openly humiliated (by doctors, nurses) because of your age” (coded as “Yes” (“Rarely”, “Often”, “Usually”, “Always”) = 1 vs. “No” (“Never”) = 0); “Do you think that your privacy was more violated because of your age compared to younger people?” (coded as “Yes” = 1 vs. “No” = 0; answer “I do not know” was eliminated from the analysis).

#### 2.2.2. Overall Measure of Age Discrimination in Healthcare

Overall measure of age discrimination in healthcare (dependent variable) was assessed by summing the “yes” answers to all nine questions, after reversing the answers to the first two reverse questions. The internal consistency of these nine statements was good, with a Cronbach’s alpha of 0.828. Discrimination was considered to have been experienced if a respondent answered positively to more than two out of nine questions/statements (coded as “Yes” = 1), whereas endorsement of two or fewer items was classified as indicating no experience of discrimination or only minimal discrimination (coded as “No” = 0). Because no validated composite measure or cut-off exists for this type of discrimination scale, the threshold was defined conceptually and empirically. This cut-off represents endorsement of at least one-third of the items, which was considered sufficient to indicate a consistent rather than incidental experience of discrimination. To evaluate the robustness of this threshold, supplementary analyses were carried out using the continuous discrimination score. The findings and overall conclusions remained unchanged, suggesting that the chosen cut-off did not affect the study’s primary results.

#### 2.2.3. Other Questions About Age Discrimination in Healthcare

During the study, respondents were also asked whether it was important for them that a doctor understood the specifics of their age (coded as “Important” = “Important”, “Very important”, “Neither important nor unimportant” and “Unimportant” = “Unimportant”, “Completely unimportant”), which determines the attitude of doctors/other healthcare professionals towards older people. In addition, five questions/statements assessed the respondents’ awareness of their rights and discrimination in healthcare, with answers “I knew”, “I did not know”. The respondents were asked whether they were aware of their rights they had as individuals and as patients separately. It also asked whether they were aware of where to file a complaint if their rights were violated in a healthcare institution, and whether they knew that they had the right to receive compensation for harm caused by healthcare professionals.

#### 2.2.4. Sociodemographic Factors

Information was collected about the sociodemographic characteristics of the participants: gender (coded as “Female” (reference group (ref)) vs. “Male”), age (grouped into five-years groups: 60–64 (ref), 65–69, 70–74, 75–79 and 80–84), marital status (coded as “Married or cohabiting” (ref) vs. “Not married” (“divorced,” “widowed,” or “single, never been married”), education (coded as “University” (ref), “Vocational/college” (“Vocational” (Grades 11–12 or gymnasium), “Special vocational”, “College”) and “Secondary or lower” (“Secondary” (8–9 grades), “Primary” (4 grades), “No education”). Evaluation of the financial status of the respondents was based on the participant’s answer to the question “Are you worried about everyday expenses?” (coded as “Good” (ref; “Sometimes”, “Never”) vs. “Poor” (“Always”, “Usually”).

#### 2.2.5. Other Factors

The health status of the participants was assessed with the question, “How do you perceive your health status?” with answers “Good” (ref; “Very good”, “Good”), “Satisfactory” and “Poor” (“Poor”, “Very poor”). The opinions of the participants were also analysed according to the type of institution in which they chose their family doctor (“Private” (ref) vs. “Public”).

### 2.3. Statistical Data Analysis

Data were recorded and analysed using the Statistical Package for Social Sciences (IBM SPSS Statistics) version 27.0. Descriptive statistics were used to summarize sample characteristics. Bivariate associations between perceived age discrimination and sociodemographic factors were examined using Chi-square tests and z tests for pairwise comparisons. To identify independent predictors of perceived age discrimination, binary logistic regression was performed, including all sociodemographic variables simultaneously in the model. The statistical significance was defined as *p* < 0.05.

### 2.4. Sample Characteristics

Responses were obtained from 492 older adults. The response rate was 37.85%. The characteristics of participants are presented in Table 1. Among the respondents, there were more females (68.9%) than males. The age of respondents varied from 60 to 84 years, with the mean age of 71.6 ± 6.6 years. More than half of the research participants (57.8%) were married or cohabiting, 42% had university education, 38.5%—vocational or college, 61.8% said they were not worried about daily expenses, 65.8% rated their current health status as satisfactory. The majority (79.6%) of the respondents chose a public primary healthcare institution.

A comparison of the sample’s age and gender structure with that of Kaunas and the national population is provided in Supplementary Table S1. The distribution of the study sample by sex and age closely resembled that of the older population in Kaunas and in Lithuania overall; therefore, the sample can be considered reasonably representative of these populations. Minor differences were observed in the youngest (60–64 years) and oldest (80–84 years) age groups, where the proportions of respondents were slightly lower than in the corresponding population groups.

## 3. Results

### 3.1. Older Adults Face the Age Discrimination in Healthcare

Table 2 demonstrates the older adults’ attitudes toward age discrimination in healthcare.

The majority of respondents (74.8%) disagreed with the statement that national health policy consistently improves the protection of older individuals’ rights in healthcare. Nearly half (42.5%) did not agree that the state guarantees equal healthcare for all age groups (both young and old), and 43.5% agreed with the statement that the older a person is, the less their healthcare and policy-related needs are met. Additionally, 42.3% believed that doctors and other healthcare professionals show less attention and care to older people. On the other hand, only 15.5% of participants reported being discouraged from undergoing procedures, surgeries, or other services due to their age, 13.3% felt openly demeaned by medical staff because of their age, and only 6.9% believed that their privacy was more likely to have been violated due to age in comparison to younger people.

It was also assessed what proportion of respondents reported experiencing age-related discrimination in healthcare (Table 2). Overall, 72% of respondents indicated experiencing discrimination in at least one of the nine assessed aspects; 55.6% endorsed at least two statements, 41.1% at least three, and one third (32.7%) endorsed four statements indicating experiences of discrimination. In the subsequent analysis, respondents who endorsed at least three out of nine statements were classified as having experienced discrimination. This threshold was selected to reflect a non-random, more systematic pattern of discriminatory experiences.

### 3.2. Factors Associated with Older Adults’ Attitudes Towards Age Discrimination in Healthcare

Table 3 presents associations between older adults’ attitudes towards age discrimination in healthcare and their sociodemographic as well as health and institutional characteristics. The respondents of older age and those reporting poorer financial and health status were more likely to believe that the state does not guarantee equal healthcare for all age groups and more frequently reported having been openly demeaned by medical staff due to their age (*p* < 0.05). Women, along with those in poorer financial and health conditions, more often agreed with the statement that the older a person is, the less their healthcare and policy needs are met. They also tended to perceive less attentiveness from medical staff and experienced longer delays in receiving help (*p* < 0.05). Older individuals with lower levels of education and those with poorer financial and health status more frequently perceived being treated worse by healthcare staff due to their age and reported feeling discouraged from using various healthcare services (*p* < 0.05). Additionally, the respondents who were registered with a general practitioner in public healthcare institutions reported experiencing poorer treatment due to their age more often than those attending private institutions (*p* < 0.05). The participants with poor financial and health status were also more likely to believe that their privacy had been compromised due to their age (*p* < 0.05). Marital status was not significantly associated with perceived age discrimination in healthcare.

Table 4 presents the associations between sociodemographic characteristics, health-related factors, and experiences of age discrimination in healthcare. Bivariate analyses (χ^2^ tests) indicated that poor self-reported health and lower financial status were significantly associated with experiencing age discrimination (*p* < 0.05). Multivariate logistic regression confirmed these findings, with poor health (OR = 4.02, 95% CI: 1.51–10.73, *p* < 0.05) and poor financial status (OR = 1.94, 95% CI: 1.16–3.25, *p* < 0.05) as independent risk factors. These results suggest that both health and economic vulnerability increase the likelihood of perceiving age-based discrimination.

### 3.3. Awareness of Human Rights

The majority of the respondents indicated awareness of their rights both as human beings (77.3%) and as patients (66.7%). Furthermore, 84.0% of the respondents agreed that the state is obligated to ensure that human rights are not violated. However, only half of the respondents reported being aware of subtle or indirect discrimination, where physicians refrain from explicitly mentioning a person’s age but nevertheless fail to deliver necessary medical services without adequate explanation (54.1%). Additionally, 49.9% recognised ageist attitudes when healthcare professionals made statements such as “What do you expect at your age?”, “There is little to hope for at this stage in life”, or “Consider whether it is worth it,” suggesting discriminatory behaviour based on age.

### 3.4. Respondents’ Perceptions of Factors Shaping Healthcare Professionals’ Attitudes Toward Older Adults

This section examines survey participants’ perceptions of the factors that influence healthcare professionals’ attitudes toward older adults, as well as how these perceptions differ according to respondents’ sociodemographic characteristics, health status, and personal experiences of discrimination.

**Figure 1:**
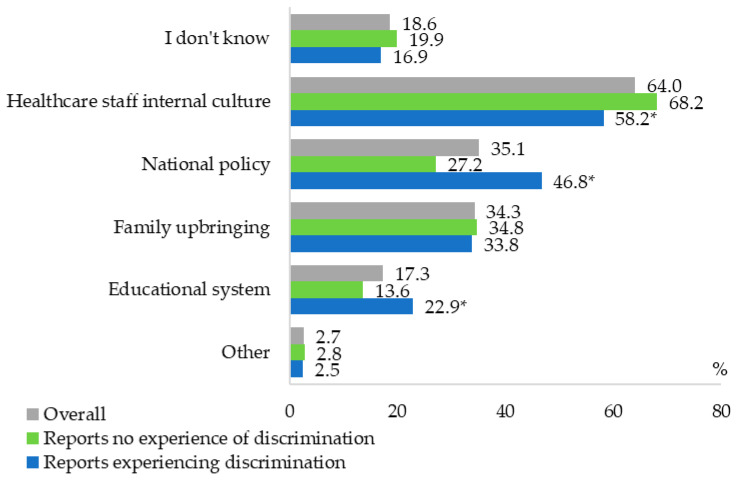
Respondents’ perceptions of factors influencing healthcare professionals’ attitudes toward older adults, overall and by experience of discrimination. * p < 0.05, compared with those reporting no experience of discrimination (z-test).

Figure 1 presents the distribution of factors identified by respondents. A substantial majority (73.3%, n = 352) agreed that it is important for physicians to understand the specific characteristics of older age. The factor most frequently perceived as shaping healthcare professionals’ attitudes was the internal culture of healthcare staff (64.0%). Approximately one-third of respondents attributed these attitudes to national policy (35.1%) or family upbringing (34.1%). Significant differences emerged depending on whether respondents had personally experienced discrimination. Individuals reporting such experiences were more likely to attribute ageist attitudes among healthcare professionals to the educational system, national policy, and internal professional culture (all *p* < 0.05), compared with respondents who had not experienced discrimination. These findings suggest that personal encounters with discrimination may heighten awareness of systemic or institutional drivers of ageism.

Perceptions also varied significantly according to education level and health status (*p* < 0.05). Respondents with higher education and those reporting good health were more likely to identify the internal culture of healthcare professionals as a key determinant of discriminatory attitudes (80.0% and 73.8%, respectively), compared with respondents with vocational/college education (55.2%), secondary or lower education (48.0%), or poorer health (48.4%). Conversely, individuals in poorer health were more likely than those in satisfactory or good health (46.9%, 36.9%, and 32.3%, respectively) to attribute ageist attitudes to state policy.

No significant associations were observed between respondents’ explanations for experiencing discrimination and their gender, age, marital status, or financial status (*p* > 0.05).

## 4. Discussion

The present study aimed to identify the frequency and most common manifestations of age-based discrimination in the Lithuanian healthcare system from the perspective of older adults. It also sought to examine how these perceptions are associated with sociodemographic characteristics, institutional culture, and national policy orientation.

The findings align with the growing evidence confirming that older people frequently experience age-based discrimination in healthcare. For example, qualitative studies describe older adults being dismissed as “attention-seeking” when they report pain and having their symptoms attributed solely to age: one Iranian study found that relatives of older adults recounted nurses refusing to believe an older person had been in severe pain. In another study, nurses reported witnessing colleagues dismiss very old patients and even ignore emergency situations involving them, sometimes resulting in delayed care and death [18]. These studies provide concrete evidence that older people are often given lower priority and even denied care. Not only do patients and providers describe dismissive or neglectful behaviour, but quantitative analyses link age bias to under-treatment, under-diagnosis and even refusal of services [19]. In the present quantitative research, the participants were considered to have experienced age-based discrimination if they agreed with at least three out of nine statements related to discriminatory attitudes or behaviours. According to this threshold, 41.1% of respondents were classified as having experienced discrimination based on age within healthcare settings (Table 2). These findings strongly confirm that older people face discrimination in healthcare settings.

Ageism in healthcare appears to arise from multiple sources. In this study, the respondents were asked what they believed to be the main factors contributing to their experience of discrimination. The most frequently selected response was the internal culture of healthcare staff (64%). This finding is consistent with other studies that emphasise the importance of healthcare professionals’ education and clinical experience in shaping their attitudes towards older people. At the individual level, providers’ personal beliefs and emotions play a role. Nurses in Tehran attributed negative care to colleagues’ intrinsic biases, noting that “some members of our healthcare team have a negative attitude towards older adult patients. They treat them as if they are children, indifferent to their personal health” [20]. Providers with high anxiety about their own aging or fear of death have been shown to hold more ageist attitudes [21]. Similarly, providers who view aging predominantly as weakness or decline, may unconsciously neglect older adults. The review of factors cited by nurses indicated that their peers often rationalised poor care by pointing to person traits: older persons were described as “needing a lot of care” and with illnesses “as if you take care of a few patients” [18], implying that the extra effort required makes them burdensome. Providers also admitted that when an older person has multiple problems or is very ill, they are more likely to be deprioritised in a busy hospital system [18].

At the system level, organisational and cultural factors also shape ageism. In the conducted study, the second most frequently selected statement was that it is state policy itself that contributes to the discrimination in healthcare (35.1%). Findings in other studies and reviews are more moderate, yet similar. Nurses reported that the culture of “no priority for older adults” and inadequate geriatric training contribute to discrimination [18]. Structural barriers–such as a lack of policies tailored to the needs of older adults, shortages of nursing staff trained in geriatric care, and cost-containment pressures–were identified as “influential factors in the discrimination against [older adults]”. Indeed, one qualitative study noted that economic and policy factors such as low insurance coverage and a lack of social support make treatment of poor older people “costly” and therefore of lower priority. For example, during the COVID-19 pandemic societal norms and some triage guidelines de-emphasised care for the very old, reinforcing stereotypes that older lives are “less important” [7]. The terror-management theory suggests such bias may be an ego-protective response to the fear of death [22]. In sum, both personal prejudice (stereotypes and negative emotions) and contextual factors (workload, a lack of resources, policy incentives) emerge as determinants of ageism in healthcare.

Consistent with these observations, certain groups of older people appear especially vulnerable. The literature indicates that socially marginalised older adults–those with lower education or income–tend to report higher levels of ageism. Although the causality is complex, one interpretation is that wealthier or better-educated older adults have more resources to conceal or manage age-related indicators (through better living conditions or knowledge) and thus experience fewer overt slights. Conversely, older people with low socioeconomic status may appear more stereotypically “old” (due to health or appearance), making them targets of bias. For example, nurses noted that old age is not pretty [23]. This suggests a bi-directional pattern: marginalized older people both perceive and attract more ageist treatment. In the present research, the respondents in poorer financial and health conditions were significantly more likely to encounter unequal treatment in healthcare, including having been openly demeaned or discouraged from services due to age (*p* < 0.05). These perceptions were also more common among older individuals, those with lower education levels, and patients attending public rather than private healthcare institutions. Unlike aforementioned study, the findings of the present research also indicate that gender plays a role: women (patients) were significantly more likely to perceive such disparities. Likewise, discrimination appears to intensify with persons’ advancing age. In the present study, older respondents were significantly more inclined to believe that the state does not guarantee equal healthcare for all age groups and reported having been openly demeaned by medical staff due to their age (*p* < 0.05). Empirical surveys find that even within the category of “older people,” the oldest old are more likely to report age-based slights and reduced clinical options. This aligns with the concept that ageism is a spectrum, worsening with age [9,24]. In line with the findings of other researchers, respondents aged 60–65 may not self-identify as old and therefore report fewer experiences of discrimination [9], whereas the very old cannot avoid either self-awareness or others’ stereotypes. Overall, the evidence indicates that age-based bias in healthcare affects all older adults but is most pronounced for the oldest olds [25].

A total of 73.3% of respondents in the present study regarded it as important for physicians to possess an understanding of the distinctive characteristics and needs associated with older age. Factors shaping health professionals’ attitudes towards older persons include both individual and experiential elements. Education and experience stand out. Across studies, healthcare workers with gerontological training or greater contact with older adults showed more positive attitudes. One systematic review found that professionals with less knowledge about aging and less clinical experience (especially in geriatric settings) consistently scored higher on measures of ageism [21]. Conversely, “gerontological education and clinical and family experience” were identified as means to “reduce ageist attitudes toward older patients” [21]. Intergenerational contact is another key factor: providers and students who express a willingness to work with older adults tend to have fewer prejudices [21]. These findings were echoed in an educational intervention study, in which combining an aging-related curriculum with clinical placements reduced nursing students’ stereotypes and prejudices [19]. Personal attitudes and emotional comfort with aging also matter. High workload and burnout can exacerbate impatience and neglect: understaffing and “a lack of personnel” make nurses more prone to ageist care patterns [21]. Similarly, institutional cues (implicit policies that reward faster throughput over thorough care, or a “youth-centric” care ethos) condition providers to allocate resources preferentially to younger people.

In the present study, an attempt was made to examine the associations between older adults’ personal characteristics, their experiences of discrimination and their perceptions of the underlying causes of age-based discrimination in healthcare. Individuals reporting discrimination were more likely than those without such experiences to attribute ageist attitudes to the education system, national policy, and the internal culture of healthcare staff (all *p* < 0.05). Perceived determinants of these attitudes also varied by respondents’ education and health status (*p* < 0.05). Participants with higher education and good health were more likely to identify the internal culture of healthcare professionals as a key driver of discrimination, compared with those with lower education or poorer health. Conversely, respondents in poorer health were more likely to attribute discrimination to state policy than those in better health. This analysis is novel in the Lithuanian context and appears relatively unexplored in other countries as well, as no quantitative studies were identified that survey older adults and analyse these associations.

Taken together, these findings both corroborate and extend existing literature on ageism. The present study supports longstanding claims (going back to Butler’s concept of “ageism” as another form of bigotry [1]) that health rights of older people are compromised by prejudice. Nevertheless, the findings of the present study—demonstrating a considerable prevalence of perceived age-based discrimination in healthcare settings—should elicit comparable concern among health policy-makers. A substantial proportion of respondents (74.8%) did not agree that national health policy consistently advances the implementation of the rights of older individuals within the healthcare system. The implications are particularly significant given that healthcare is a critical domain where equity and non-discrimination must be ensured to uphold the fundamental right to health for all individuals, regardless of age. Almost half of the respondents (42.5%) disagreed with the notion that the state ensures equal access to healthcare across all age groups. Furthermore, 43.5% agreed that the older an individual is, the less adequately their healthcare needs are addressed. Similarly, 42.3% indicated that medical practitioners and other healthcare personnel tend to demonstrate reduced attentiveness and concern towards older patients. Thus, ageism in healthcare warrants urgent attention as a social justice and equity issue.

### 4.1. Implications

Clinicians and policymakers must recognise that older people are not receiving equitable care, which can worsen health outcomes and violate principles of justice. Health systems should consider strategies such as geriatric training for all providers, the inclusion of older adult representatives in care planning, and the implementation of anti-discrimination safeguards (e.g., monitoring treatment decisions for age bias). The findings suggest that more geriatric clinical experiences and explicit discussion of age stereotypes should be integrated into curricula. At the policy level, guidelines for resource allocation (e.g., (Intensive care unit (ICU) beds, transplants, screening) should be reviewed to ensure they do not implicitly discriminate by age. The evidence of social gradients means that outreach to less-educated, low-income elders is especially warranted, perhaps through community health workers or targeted subsidies. Finally, it is important to raise awareness–among health professionals and the public–that ageism exists and damages the health of older people. Studies cited here recommend culture change measures (training, awareness campaigns) to counteract “ageist assumptions that every ailment is due to old age” [20].

### 4.2. Strengths and Limitations

Many existing studies rely on self-report surveys or small qualitative samples, which can limit generalisability. The sample size in the present study is indeed sufficient. According to the Raosoft sample size calculator, a minimum 387 responses was needed. As 492 questionnaires were completed, the achieved sample size exceeds the recommended threshold. However, there are certain limitations.

The cross-sectional design does not allow for causal inference, and the observed age differences may partially reflect cohort effects. The lack of standardized instruments for assessing ageism represents an additional limitation. At the time of the study, there were no existing measures of ageism in the healthcare context.

Furthermore, the data were collected several years ago, and the prevalence of ageism may have shifted due to the COVID-19 pandemic and other societal changes. One of the limitations of this study is that the sample represents only the urban population of Kaunas city and may not fully reflect the experiences of older adults in rural areas or smaller towns. Therefore, caution is advised when generalizing the findings to the entire older population in Lithuania. Nevertheless, Kaunas is the second-largest city in the country, and the study offers meaningful insights into the challenges faced by older adults in urban healthcare settings.

An additional limitation is that very old adults (85+ years) are not included in the study sample, although they may be particularly vulnerable to ageism in healthcare settings.

### 4.3. Future Directions

Further research is needed to fill existing gaps. Controlled studies of anti-ageism training, continuing professional education, and intergenerational practice models are needed to clarify what truly changes provider behaviour. More work on structural factors–such as how hospital policies, reimbursement rules, or quality metrics might perpetuate age bias–could inform system-level reforms. Quantitative studies with larger and more representative patient samples would help quantify how ageism varies by country, care setting, and specialty. Longitudinal studies could track whether changing demographics (e.g., aging population) or major events (like pandemics) alter patterns of age-based care. It would also be valuable to examine intersectionality more deeply; for instance, how race, gender, disability, and socioeconomic status interact with age to shape patients’ treatment. Finally, legal and rights-based research–such as that exemplified by “Never too old for health and human rights?” [26]—could investigate how age discrimination in healthcare breaches older persons’ rights frameworks and potentially drives policy change.

In conclusion, the found evidence confirms that ageism is a real and damaging force in healthcare systems. Older people–especially those who are poorer, less educated, or very old–often receive suboptimal treatment due to stereotypes, prejudice, and systemic bias. Addressing these issues will require concerted effort: educating providers, monitoring of care practices, and fostering broader cultural shifts in how society values older people. The findings of this research underscore the importance of explicitly acknowledging ageism in healthcare provision, health policy, and practice, so that advancing age no longer becomes a barrier to receiving the care every person deserves.

## 5. Conclusions

This study provides empirical evidence that perceived age-based discrimination is a significant concern among older individuals in the Lithuanian healthcare system. Nearly half of the respondents (41.1%) reported experiencing age-related discrimination, with the highest prevalence observed among individuals of older age, lower educational attainment, and poorer financial and health conditions. Women and patients receiving care in public healthcare institutions were also more likely to perceive discriminatory treatment.

The findings suggest that perceptions of ageism in healthcare are strongly influenced not only by individual characteristics but also by broader systemic and institutional factors. The internal culture of healthcare professionals emerged as the most frequently cited determinant of discriminatory attitudes (64.0%), while over a third of participants also identified national policy and family upbringing as influential factors. The respondents with higher education and good health were more likely to state that discrimination stem from the internal culture of healthcare professionals rather than external factors such as state policy. Furthermore, a considerable share of respondents recognized indirect or subtle forms of ageism, including dismissive remarks and unexplained denial of care.

Although most respondents were aware of their rights as human beings and patients, a substantial proportion expressed scepticism regarding the effectiveness of national policies in protecting older adults’ rights in healthcare. This highlights a disconnect between formal policy commitments and patients’ actual experiences.

These results underline the urgent need for multi-level interventions to address ageism in healthcare, including improvements in geriatric training, strengthening of institutional cultures, and policy reforms that explicitly safeguard the rights and dignity of older individuals.

## Figures and Tables

**Table 1:** Characteristics of the sample (N = 492).

Characteristics	%	N
**Gender**		
Female	68.9	337
Male	31.1	152
All	100	489
**Age groups in years**		
(min 60, max 89, mean 71.6, SD 6.6)		
60–64	17.7	86
65–69	22.8	111
70–74	24.4	119
75–79	21.4	104
80–84	13.8	67
All	100	487
**Marital status**		
Married or cohabiting	57.8	282
Not married	42.2	206
All	100	488
**Education**		
University	42.0	204
Vocational/college	38.5	187
Secondary or lower	19.5	95
All	100	486
**Perceived financial status** (are you worried about everyday expenses?)		
Good (sometimes/never)	61.8	302
Poor (always/usually)	38.2	187
All	100	489
**Perceived health (self-reported current health status)**		
Good (very good/good)	21.1	103
Satisfactory	65.8	322
Poor (poor/very poor)	13.1	64
All	100	489
**Type of primary healthcare institution**		
Public	79.6	382
Private	20.4	98
All	100	480

**Table 2:** Respondents’ attitudes towards age discrimination in healthcare.

Statements		All Responses ^a^	Yes	No
		% (n)	% (n)	% (n)
1R	The state guarantees equal healthcare for all age groups (both young and older?)	93.7 (475)	57.5 (180)	42.5 (133)
2R	National health policy consistently improves the protection of rights of the older and elderly individuals in healthcare	96.1 (473)	25.2 (79)	74.8 (234)
3	The older a person is, the less their needs are met in healthcare and healthcare policy	97.2 (478)	43.5 (208)	56.5 (270)
4	The older a person is, the less attention and care are provided by doctors and other healthcare professionals	97.0 (477)	42.3 (202)	57.7 (275)
5	The older a person is, the more likely healthcare professionals are to delay providing assistance	97.0 (477)	25.8 (123)	74.2 (354)
6	You have felt that staff in healthcare institutions treated you worse due to your age	92.5 (455)	34.7 (158)	65.3 (297)
7	You have been discouraged from undergoing procedures, surgeries, or other services because of your age	82.5 (406)	15.5 (63)	84.5 (343)
8	You have been openly demeaned due to your age (by doctors or nurses)	90.9 (447)	13.3 (57)	87.0 (381)
9	Your privacy was more likely to have been compromised due to your age compared to younger people	90.9 (447)	6.9 (27)	93.1 (365)
Respondents who agreed with at least one statement indicating discrimination		99.4 (489)	72.0 (352)	28.0 (137)
Respondents who agreed with at least two statements indicating discrimination		99.4 (489)	55.6 (272)	44.4 (217)
**Respondents who agreed with at least three statements indicating discrimination**		**99.4** **(489)**	**41.1** **(201)**	**58.9** **(288)**
Respondents who agreed with at least four statements indicating discrimination		99.4 (489)	32.7 (160)	67.3 (329)

R—reverse statement; ^a^—respondents who did not answer the question and who answered “do not know” or “do not evaluate” were excluded; cell shading indicates the level of experienced discrimination, with dark green representing the lowest and dark orange the highest level; lighter shades indicate intermediate levels; the row in bold indicates the criterion used to classify respondents as having experienced discrimination in the subsequent analysis.

**Table 3:** Respondents’ attitudes towards age discrimination in healthcare depending on various factors.

Characteristics	Statements (Perceived Discrimination, %)								
	1	2	3	4	5	6	7	8	9
**Gender**									
Female	42.9	73.6	**46.8 ^a^**	**45.4 ^a^**	**28.2 ^a^**	34.2	17.2	14.2	7.9
Male	40.2	76.1	**35.8 ^b^**	**34.5 ^b^**	**19.6 ^b^**	35.6	14.8	10.6	4.8
**Age groups in years**									
60–64	**34.5 ^a^**	72.7	43.5	47.1	**35.3 ^a^**	**25.0 ^a^**	16.0	**8.6 ^a^**	8.1
65–69	**29.9 ^a^**	77.3	38.0	35.2	24.8	**28.3 ^a^**	**9.5 ^a^**	**7.9 ^a^**	2.4
70–74	44.2	71.2	44.2	43.4	**21.6 ^b^**	33.6	17.6	13.0	9.1
75–79	**56.7 ^b^**	78.8	45.5	40.6	25.7	**45.7 ^b^**	14.1	18.1	6.1
80–84	42.5	71.4	47.0	46.2	**19.7 ^b^**	**44.1 ^b^**	**25.5 ^b^**	**21.6 ^b^**	9.8
**Marital status**									
Married or cohabitants	40.5	76.2	46.0	42.3	25.6	36.2	15.7	11.4	5.4
Not married	44.2	71.9	40.1	41.8	25.6	32.8	15.4	15.5	8.9
**Education**									
University	39.7	76.6	42.4	38.4	22.7	**27.1 ^a^**	**11.2 ^a^**	9.5	4.1
Vocational/college	40.2	71.6	44.9	46.3	29.4	**39.8 ^b^**	16.9	14.6	9.7
Secondary or lower	50.9	74.6	42.9	41.8	24.2	**42.4 ^b^**	**23.7 ^b^**	17.4	8.0
**Financial status**									
Good	**36.6 ^a^**	70.9	**37.4 ^a^**	**36.5 ^a^**	**20.1 ^a^**	**28.8 ^a^**	**10.4 ^a^**	**9.3 ^a^**	**4.0 ^a^**
Poor	**50.0 ^b^**	79.7	**53.0 ^b^**	**50.8 ^b^**	**34.4 ^b^**	**44.0 ^b^**	**23.7 ^b^**	**19.3 ^b^**	**11.8 ^b^**
**Self-health**									
Good	**26.1 ^a^**	68.2	**34.0 ^a^**	**35.0 ^a^**	**20.8 ^a^**	**16.5 ^a^**	**5.2 ^a^**	**4.5 ^a^**	**3.7 ^a^**
Satisfactory	**45.3 ^b^**	75.4	**43.6 ^a^**	41.8	25.2	**36.2 ^b^**	**16.5 ^b^**	**14.4 ^b^**	**6.3 ^a^**
Poor	**52.5 ^b^**	80.5	**57.1 ^b^**	**54.0 ^b^**	**34.9 ^b^**	**54.1 ^c^**	**24.6 ^b^**	**19.3 ^b^**	**14.3 ^b^**
**Type of the institution**									
Public	40.1	73.3	43.7	42.7	25.2	**37.6 ^a^**	16.5	12.7	7.1
Private	50.8	80.0	40.4	34.9	26.3	**23.9 ^b^**	9.8	12.2	4.7

1—The state guarantees equal healthcare for all age groups (both young and older?), 2—National health policy consistently improves the protection of rights of the older and elderly individuals in healthcare, 3—The older a person is, the less their needs are met in healthcare and healthcare policy, 4—The older a person is, the less attention and care are provided by doctors and other healthcare professionals, 5—The older a person is, the more likely healthcare professionals are to delay providing assistance, 6—You have felt that staff in healthcare institutions treated you worse due to your age, 7—You have been discouraged from undergoing procedures, surgeries, or other services because of your age, 8—You have been openly demeaned due to your age (by doctors or nurses), 9—Your privacy was more likely to have been compromised due to your age compared to younger people; ^abc^—different letters show statistically significant differences between groups (for example, a statistically significantly higher proportion of women than men agreed with statements 3, 4, and 5; *p* < 0.05; z test); statistically significant differences are shown in bold.

**Table 4:** Comparison of perceived discrimination between (sociodemographic) characteristics.

Characteristics	Experienced Discriminatory Behaviour		OR	95% CI
	Yes (n = 201)	No (n = 288)		
	% (n)	% (n)		
**Gender**				
Female	43.0 (144)	57.0 (191)	1	
Male	35.8 (54)	64.2 (97)	1.018	0.543–1.909
	χ^2^ = 2.250; df = 1; *p* = 0.134		*p* = 0.954	
**Age groups**				
60–64	44.2 (38)	55.8 (48)	1	
65–69	34.2 (38)	65.8 (73)	0.560	0.246–1.272
70–74	40.7 (48)	59.3 (70)	0.723	0.334–1.567
75–79	41.7 (43)	58.3 (60)	0.555	0.244–1.262
80–84	45.5 (30)	54.5 (36)	0.747	0.290–1.929
	χ^2^ = 3.021; df = 4; *p* = 0.554		*p* = 0.607	
**Marital status**				
Married or cohabiting	43.0 (120)	57.0 (159)	1	
Not married	37.9 (78)	62.1 (128)	0.717	0.412–1.248
	χ^2^ = 1.299; df = 1; *p* = 0.254		*p* = 0.240	
**Education**				
University	36.8 (75)	63.2 (129)	1	
Vocational/college	41.6 (77)	58.4 (108)	0.781	0.319–1.911
Secondary or lower	47.9 (45)	52.1 (49)	1.299	0.383–4.407
	χ^2^ = 3.374; df = 2; *p* = 0.185		*p* = 0.510	
**Financial status**				
Good	**34.7 (104) ^a^**	**65.3 (196) ^a^**	1	
Poor	**50.5 (94) ^b^**	**49.5 (92) ^b^**	**1.944**	1.163–3.249
	χ^2^ = 11.979; df = 1; *p* = **0.001**		*p* = **0.011**	
**Self-health**				
Good	**31.3 (32) ^a^**	**33.0 (34) ^a^**	1	
Satisfactory	**40.4 (129) ^a^**	**29.2 (93) ^b^**	1.856	0.825–4.177
Poor	**57.8 (37) ^b^**	**15.6 (10) ^b^**	**4.019**	1.506–10.728
	χ^2^ = 11.730; df = 2; *p* = **0.003**		*p* = **0.016**	
**Type of the institution**				
Private	36.1 (35)	62 (63.9)	1	
Public	42.8 (163)	57.2 (218)	1.080	0.565–2.063
	χ^2^ = 1.430; df = 1; *p* = 0.232		*p* = 0.817	

χ^2^ tests indicate bivariate associations between discrimination experience and sociodemographic characteristics. OR and 95% CI are derived from multivariate logistic regression including all variables simultaneously (Cox & Snell R Square = 0.084). Different letters (a, b) indicate statistically significant differences between two groups based on z-test for column proportions (for example, respondents with poor financial status reported discrimination significantly more often than those with good financial status (50.5% vs. 34.7%, z-test, *p* < 0.05); statistically significant differences and OR are shown in bold.

## Data Availability

The datasets presented in this article are not readily available because the data are part of an ongoing study and PhD dissertation. Requests to access the datasets should be directed to corresponding author.

## Supplementary Materials

The following supporting information can be downloaded at: https://www.mdpi.com/article/10.3390/healthcare13243192/s1, Table S1. The structure of Kaunas residents in 2019 and the study sample by age and gender.

## Abbreviations

The following abbreviations are used in this manuscript:

WHO	World Health Organization
U.S.	United States
ECHR	European Court of Human Rights
EU	European Union
DT	Digital Technologies
IBM SPSS	International Business Machines–Statistical Package for the Social Sciences
ICU	Intensive Care Unit
